# Job stress and burnout in the care staff of Leros PIKPA Asylum 25 years after the first Deinstitutionalisation and Rehabilitation Greek Project

**DOI:** 10.1192/pb.bp.115.052258

**Published:** 2016-12

**Authors:** Anastasia Bougea, Manolis Kostas Kleisarchakis, Nikolaos Spantideas, Panagiota Voskou, Thomas Thomaides, George Chrousos, Sophia Andreas Belegri

**Affiliations:** 1 First Department of Neurology, School of Medicine, University of Athens, Athens, Greece; 2 Primary Education Program, Heraklion, Crete, Greece; 3 Athens Speech and Language Institute, Greece; 4 Benakeio and Korgialeneio Hospital, Athens, Greece; 5 First Department of Pedicatrics, School of Medicine, University of Athens, Athens, Greece; 6 Social Service, Monemvasia, Lakonia, Greece

## Abstract

**Aims and method** To identify correlates between burnout and job stress of care staff at Leros PIKPA Asylum. Forty-nine asylum employees were assessed by Maslach's Burnout Inventory, a sociodemographic questionnaire, the Perceived Stress Scale and the Job Content Questionnaire.

**Results** Emotional exhaustion is related negatively to social support (*P* = 0.010, *r* = −0.362). Lack of job achievements is related positively to overall job responsibility (*P* = 0.040) and negatively to lack of job satisfaction (*r* = −0.430). Depersonalisation was negatively associated with support from superiors (*P* = 0.036). Employees with high levels of perceived stress reported higher levels of fatigue (*P* = 0.050). Positive associations of perceived stress with depression (*P* = 0.011) and sleep problems (*P*<0.001) were also detected. Positive correlation was found between monthly salary and lack of sense of personal achievement (*P* = 0.020).

**Clinical implications** It is necessary to address these issues through staff education and stress management.

Burnout syndrome comprises emotional exhaustion, depersonalisation and a reduced sense of personal accomplishment and is a major problem for health professionals.^1,2^ A high degree of burnout is very common among nursing staff.^3–5^ According to the demands-control model of Karasek, the job content reveals that certain sources of job stress, high job demands and low level of control by the employee over their work are responsible for the stress experienced in the workplace.^4^

The staff of psychiatric hospitals and institutions are particularly exposed to stress factors which are related to the nature of care they provide, as well as the organisational frame of the psychiatric nursing institutions. The emotional stress of taking care of patients with psychiatric problems, workload, the feeling of control at work and the social and economic rewards associated with work are factors related to job stress and professional exhaustion.^5,6^ Specifically, staff who have close, face-to-face interactions with patients and those with longer job tenure in mental health were more likely to develop burnout. A study of 510 psychiatric workers in 28 different units found that high levels of emotional exhaustion and depersonalisation were correlated with negative attitudes (e.g. distant, rejecting) toward patients on their ward.^7^ Another study of 93 nurses from 11 acute adult mental health wards showed that difficult or demanding patients were the most stressful aspect of their job for unqualified staff.^8^ Approximately half of all nursing staff showed signs of high burnout in terms of emotional exhaustion. Higher stressor scores were associated with higher levels of depersonalisation. Reduced level of support and increased administrative pressures were leading to an increase in stress and burnout.^9^

The full extent of the problems arising from the provision of institutionalised care only came to light in the early 1990s, when the first deinstitutionalisation project in Greece for people with intellectual, physical, motor and other disabilities was implemented. The Leros Medical-Social Welfare (formerly PIKPA) Asylum Deinstitutionalisation and Rehabilitation Project concerned an institution for children with special needs on the Greek island of Leros. This project was carried out under the direction of J. Tsiantis, professor of child psychiatry. At the beginning of the project, there were 165 residents aged 8–46, out of whom 32 (20%) were children and adolescents up to 18 years old. Living conditions and sanitation within the setting were found to be degrading and unacceptable, nutrition was poor, residents were packed into 40-bed wards, and the number of beds was insufficient. There was also a lack of trained personnel, a lack of medical, nursing and physiotherapeutic care, a total absence of special equipment or educational materials, and an extensive use of violence and physical restraint by the personnel. At the end of the intervention project, improvements in the care system and patients' quality of life in the Leros PIKPA Asylum were obvious. Following training, the care staff changed their unacceptable care methods and negative attitude towards people with disabilities.^10^ However, since then no other intervention effort was pursued in Leros PIKPA.

Twenty-five years after the first deinstitutionalisation, the aim of the present study was to investigate the correlation between job content and burnout syndrome among the staff of Leros PIKPA Asylum. Previous studies showed the significance of the application of intervention programmes for deinstitutionalisation and rehabilitation,^10–12^ as well as the need for supporting employees with high levels of stress and difficulties in their workplace.^12^

## Method

### Study design and procedure

This is a cross-sectional study that took place between August and November 2013 in Leros PIKPA Asylum on the Greek island of Leros. It was conducted with the help of nursing managers at the four sectors of the asylum. The head nurse of each sector distributed the questionnaires and the study included asylum employees (nursing and administrative staff). To maintain the privacy of the participants, all questionnaires were delivered to the main investigator in closed envelopes. The inclusion criteria were permanent employment in the asylum and no pre-existing mental illness.

The study was approved by the PIKPA Hospital Ethics Committee (trial registration number UoAMedPR-4716-180211-19) and complied with the 2013 World Medical Association Declaration of Helsinki. All participants were given a letter containing information about the study's aims and procedures. The voluntary nature of participation and anonymity were emphasised in the informed consent form.

### Measures

All participants received four questionnaires: a sociodemographic questionnaire, the Maslach Burnout Inventory (MBI), the Job Content Questionnaire (JCQ) and the Perceived Stress Scale (PSS).^13–15^

The sociodemographic questionnaire included the following: gender, age, level of education, marital status, number of children (if any), work type, work hours, monthly salary, previous experience at the asylum and shift flexibility (ability to change shifts).

The MBI,^13^ validated in Greek,^2^ consists of 22 items distributed across the 3 dimensions of burnout: emotional exhaustion (9 items), depersonalisation (5 items) and the feeling of personal accomplishment (8 items). Participants rated the frequency of burnout symptoms on a seven-point Likert scale, ranging from 0 (‘never happens to me’) to 7 (‘it happens to me every day’). Depersonalisation represents the interpersonal burnout dimension and refers to negative and cynical confrontation with service recipients (patients). A typical example of such behaviour is an inclination to refer to patients not by name but by their bed or room number, or their illness. Cronbach's alpha coefficient was 0.838 for professional exhaustion, 0.902 for emotional exhaustion, 0.820 for personal attainment and 0.508 for depersonalisation.

The JCQ^14^ was designed to measure the ability to make decisions at work, work demands (psychological, physical), job insecurity, coworker and supervisor support, and quality of life. The employees' quality of life includes the following subcategories: lack of job satisfaction, depressive emotions, psychosomatic stress (fatigue, perspiration, and decreased appetite and eating) and sleep disorders, as well as complex psychosomatic stress (combined stress of the above factors). The participants were called to indicate on a four-level scale whether they agreed with the statements (‘totally disagree’, ‘disagree’, ‘agree’, ‘totally agree’). The psychometric abilities of this questionnaire have been validated in a Greek population;^15^ JCQ Cronbach's alpha was 0.878.

The PSS^16^ has been validated in the Greek population by Andreou *et al*.^17^ Seven out of the fourteen items of the PSS-14 are considered negative and the remaining seven positive. The negative part is intended to assess lack of control and negative affective reactions, while the positive elements measure the degree of ability to cope with existing stressors. Each item was rated on a 5-point Likert scale (ranging from 1 ‘never’ to 5 ‘very often’). Total scores (ranging from 0 to 56) were calculated after reversing the positive items' scores and then summing up all scores. PSS Cronbach's alpha was 0.803.

### Statistical analysis

Continuous variables are presented with mean and standard deviation. Quantitative variables are presented with absolute and relative frequencies. Pearson's χ^2^-test and Fisher's exact test were used for the comparison of frequencies between two subgroups. Where dependent variables were continuously scaled, we performed either a parametric independent samples *t*-test, if assumptions of normality and/or homogeneity of variance held, or a non-parametric Mann-Whitney U test (for the difference of mean values between two groups) and the control of Kruskal-Wallis (for the difference between mean values of more than two groups), if the assumptions were violated. The Kolmogorov-Smirnov test or the Shapiro-Wilk test were used for assessment of normality. Spearman's correlation coefficient was used to explore the association of two continuous variables. All *P*-values reported are two-tailed. Statistical significance was set at 0.05 and analyses were conducted using the SPSS statistical software (version 19.0).

## Results

Sixty employees of Leros PIKPA were invited to participate in this study; 49 agreed and subsequently completed the questionnaires. The basic demographic characteristics of the participants are presented in Table 1.

**Table 1:** Demographic characteristics of participants

Gender, *n* (%)	
Male	13 (26.53)
Female	36 (73.47)

Age, years: mean (range)	48.30 (34–65)

Marital status, *n* (%)	
Single	3 (6.12)
Married	38 (77.55)
Divorced	4 (8.16)
Widowed	4 (8.16)

Education, *n* (%)	
Primary	24 (48.98)
High school^a^	7 (14.29)
Lyseum^b^	4 (8.16)
Vocational training	8 (16.33)
University	5 (10.20)
Master/PhD	1 (2.04)

Working staff, *n* (%)	
Nurses	14 (28.57)
Chefs	2 (4.08)
Medical assistants	28 (57.14)
Administrative staff	5 (10.20)

Years worked in asylum, mean (s.d.)	20 (6.90)

Total years worked, mean (s.d.)	23.60 (8.04)

Hours worked per week, mean (s.d.)	39.60 (2.87)

Job schedule, shifts: *n* (%)	
Morning	18 (36.73)
Afternoon	1 (2.04)
Alternative without night	9 (18.37)
Alternative with night	21 (42.86)

Monthly salary, €: *n* (%)	
<1000	15 (30.61)
1000–1500	33 (67.35)
1500–2000	1 (2.04)

Possibility to change shifts, *n* (%)	
Difficult	7 (14.29)
Only in exceptional cases	15 (30.61)
Flexible schedule	27 (55.10)

a. Compulsory education from 13 to 16 years of age.

b. 16 to 18 years of age.

From the statistical analysis of the demographic characteristics of the employees (Table 2a, 2b & 2c), it emerged that higher salaries are correlated with older age as well as years of work. The mean values of years of work at the institution and total years of work were higher in male employees in relation to females (t = 3.605, *P*<0.001).

**Table 2a:** Comparison of demographic characteristics of employees: differences per gender

Demographics	Men	Women	Statistical test	*P^a^*
Salary, €: *n* (%)				
<1000	3 (6.12)	12 (24.48)		
1000–1500	9 (18.36)	24 (48.98)	χ^2^ = 0.665	0.318
1500–2000	1 (2)	0		

Education, *n* (%)				
Primary	9 (69.23)	15 (41.67)		
High school^b^	1 (7.69)	6 (16.67)		
Lyseum^c^	2 (15.38)	2 (5.56)	*z* = 1.732	0.083
Vocational training		8 (22.22)		
University	1 (7.69)	4(11.11)		
Master/PhD		1 (2.78)		

Hours worked per week, mean (range)	39.84 (38–40)	39.47 (30–48)	*t* = 0.397	0.692

Marital status, *n* (%)				
Single	11 (84.62)	3 (8.33)		
Married	2 (15.38)	27 (75.00)	χ^2^ = 0.046	0.829
Divorced		2 (5.56)		
Widowed		4 (11.11)		

Job schedule, shifts: *n* (%)				
Morning	4 (30.77)	14 (38.89)		
Afternoon	1 (7.69)		χ^2^ = 0.101	0.751
Alternative shift without night	2 (15.38)	7 (19.44)		
Alternative shift with night	6 (46.15)	15 (41.67)		

Working staff, *n* (%)				
Nurses	2 (15.38)	12 (33.33)		
Chefs		2 (5.56)	χ^2^ = 0.905	0.341
Medical assistants	10 (76.92)	18 (50.00)		
Administrative staff	1 (7.69)	4 (11.11)		

Years worked in asylum, mean (s.d.)	26 (2.70)	18.80 (6.96)	*t* = 3.605	< 0.001

Total years worked, mean (s.d.)	31.30 (7.17)	20.80 (6.40)	*t* = 4.914	< 0.001

a. *P*<0.05 significant.

b. Compulsory education from 13 to 16 years of age.

c. 16 to 18 years of age.

**Table 2b:** Comparison of demographic characteristics of employees: differences in salary and years worked in the asylum per age

	Age, years				
	< 45	45–55	>55	Statistical test	*P*
Salary, €: *n* (%)				χ^2^ = 9.908	0.007
< 1000	2 (4.08)	7 (14.28)	6 (12.24)		
1000–1500	13 (26.53)	20 (40.81)	0		
1500–2000	0	1 (2.04)	0		

Years worked in asylum, mean (s.d.)	16.80 (6.70)	21.22 (6.14)	27.14 (5.00)	χ^2^ = 10.094	0.006

**Table 2c:** Comparison of demographic characteristics of employees: differences in salary per educational level

Salary, €: *n* (%)	Primary	High school	Lyseum	Technical institution	University	Master/PhD	Statistical test	*P*
<1000	10 (20.4)	2 (4)	1 (2)	0	2 (4)	0	χ^2^ = 3.254	0.660
1000–1500	14 (28.6)	5 (20.2)	3 (6.12)	8 (16.3)	2 (4)	1 (2)		
1500–2000	0	0	0	0	1 (2)	0		

The correlations of job content with the three dimensions of professional burnout showed a statistically significant relation of the emotional exhaustion dimension of burnout to the parameters associated with psychological demands and stress factors of work in total (Table 3). Low emotional exhaustion was reported for nursing staff with coworker and supervisor support. The dimension of lack of personal achievements has a significant statistical difference in the two categories of formal authority (yes/no) (*P* = 0.04), and employees with low formal authority have an increased score in the dimension of lack of personal achievements. High levels of depersonalisation are correlated with lack of supervisor support (*P* = 0.036).

**Table 3:** Correlations between job content scores (Job Content Questionnaire) and burnout

	Burnout		
Job content	Emotional exhaustion	Personal accomplishment	Depersonalisation
Skill utilisation	*r* = −0.070	*r* = 0.090	*r* = −0.274
	*P* = 0.628	*P* = 0.729	*P* = 0.056

Skill discretion^a^	*r* = −0.137	*r* = 0.139	*r* = −0.261
	*P* = 0.346	*P* = 0.340	*P* = 0.069

Decision authority	*r* = −0.096	*r* = 0.042	*r* = −0.191
	*P* = 0.510	*P* = 0.772	*P* = 0.187

Decision scope	*r* = −0.080	*r* = 0.050	*r* = −0.218
	*P* = 0.581	*P* = 0.730	*P* = 0.132

Psychological demand	***r* = 0.484**	*r* = 0.151	*r* = 0.144
	*P*<0.001	*P* = 0.297	*P* = 0.322

Job insecurity	*r* = −0.193	*r* = −0.164	*r* = −0.124
	*P* = 0.183	*P* = 0.259	*P* = 0.393

Working hours/week	*r* = −0.004	*r* = 0.133	*r* = −0.098
	*P* = 0.977	*P* = 0.360	*P* = 0.502

Stressors (total)	***r* = 0.459**	*r* = 0.112	*r* = 0.135
	*P*<0.001	*P* = 0.443	*P* = 0.352

Formal authority (Yes/No)	*P* = 0.266	*P* = 0.040	*P* = 0.950

Participation in trade union (Yes/No)	*P* = 0.492	*P* = 0.889	*P* = 0.108

Coworker support	***r* = −0.332**	*r* = −0.184	*r* = −0.165
	*P* = 0.019	*P* = 0.205	*P* = 0.256

Supervisor support	***r* = −0.296**	*r* = −0.029	***r* = −0.300**
	*P* = 0.038	*P* = 0.839	*P* = 0.036

Social support	***r* = −0.362**	*r* = −0.168	*r* = −0.266
	*P* = 0.010	*P* = 0.246	*P* = 0.063

Fatigue (strongly disagree /disagree/strongly agree/agree)	*P* = 0.596	*P* = 0.429	*P* = 0.432

*r*, Pearson correlation coefficient.

*P*<0.05 significant.

Significant scores are shown in bold type.

a. Skill discretion refers to the degree to which the job involves a variety of tasks, low levels of repetitiveness, occasions for creativity and opportunities to learn new things and develop special abilities.

The correlations of the employees' quality of life with the three dimensions of professional burnout and the perceived stress are detailed in Table 4. Emotional exhaustion was significantly correlated with psychosomatic stress (*r* = 0.381, *P*<0.001), sleep disorders (*r* = 0.469, *P*<0.001) and complex psychosomatic stress (*r* = 0.525, *P*<0.001). The lack of personal achievements is significantly related to depressive feelings (*r* = −0.283, *P* = 0.048) and lack of work satisfaction (*r* = −0.430, *P* = 0.002). Employees with high levels of perceived stress present with depressed emotion (*r* = 0.358, *P* = 0.011) and sleep disorders (*r* = 0.633, *P*<0.001), as well as psychosomatic (*r* = 0.480, *P*<0.001) and complex psychosomatic stress (*r* = 0.609, *P*<0.001).

**Table 4:** Job Content Questionnaire (JCQ): correlation with perceived stress scale and burnout

		Burnout		
JCQ	PSS	Emotional exhaustion	Personal accomplishment	Depersonalisation
Job dissatisfaction	*r* = 0.225	*r* = 0.133	***r* = −0.430**	*r* = 0.116
	*P* = 0.119	*P* = 0.362	*P* = 0.002	*P* = 0.424

Depression	***r* = 0.358**	***r* = 0.495**	***r* = −0.283**	*r* = 0.136
	*P* = 0.011	*P*<0.001	*P* = 0.048	*P* = 0.352

Psychosomatic stress (fatigue, sweating, irritability, decreased appetite)	***r* = 0.480**	***r* = 0.381**	*r* = −0.231	*r* = 0.007
	*P*<0.001	*P* = 0.006	*P* = 0.110	*P* = 0.960

Sleep disorders	***r* = 0.633**	***r* = 0.469**	*r* = −0.002	*r* = 0.129
	*P*<0.001	*P*<0.001	*P* = 0.987	*P* = 0.374

Complex psychosomatic stress (combined score of the above factors)	***r* = 0.609**	***r* = 0.525**	*r* = −0.218	*r* = 0.098
	*P*<0.001	*P*<0.001	*P* = 0.132	*P* = 0.502

PSS, Perceived Stress Scale; *r*, Pearson correlation coefficient

*P*< 0.05 significant.

Significant scores are shown in bold type.

## Discussion

This is the first study to assess the relation between professional burnout and work stress in the Leros PIKPA Asylum. Our main findings were significant positive correlations between emotional exhaustion and psychological demands of work-related life stressors, psychosomatic stress and complex psychosomatic stress.

It seems that psychological demands due to the nature of work at Leros PIKPA may be responsible for emotional exhaustion among the nursing staff.^18^ Furthermore, stressors that manifest with the psychological demands of work combined with job insecurity lead to the emotional exhaustion of the employees, since the workload is not matched by rewards such as a stable workplace environment and potential for career advancement.^19^ The expression of psychosomatic symptoms in employees with emotional exhaustion is interpreted by the incidence of intense stress at work with mental and physical health consequences.^20,21^ A study by Kozak *et al* points in the same direction.^22^ It illustrates that the negative impact of physical and psychological exhaustion on employees providing care services to people with cognitive disorders in Germany influences their health status, while being responsible for the reduction of satisfaction derived from work and life.

Social support (from colleagues and supervisors) may be a protective factor against the negative consequences of the demands of work and subsequent emotional exhaustion.^23,24^ According to the model of demands-control-support in the work environment,^25^ support at work can promote the good health of employees and may be proposed as an element of interventional programmes for coping with stress.^26^

In the present study, the dimension of professional burnout that evaluates the employee's lack of personal achievements shows a statistically significant difference between participants with or without formal authority. It is possible that employees with formal authority have the feeling of control over their work in contrast with those without such authority who lack work control, a fact that can also be reinforced by their lower position in the work hierarchy.

The contradictory finding of a negative correlation between the lack of personal achievements and experience of depressive feelings is probably due to intermediate variables which interfere with the above relations, such as work object, age, previous work experience, education, economical gain, and position in work hierarchy.^27^ A study among nursing staff in China showed that employees with a high educational level experienced more severe depression and job dissatisfaction than those with a lower level of education.^28^ Depersonalisation also constitutes a significant dimension of professional burnout among employees having direct contact with patients presenting multiple problems, such as those at PIKPA Asylum.^29^ Supervisor support seems to have a negative impact in terms of employee depersonalisation, since the majority have received a basic level of education and do not receive any other kind of supervision in regard to patient care.

‘Perceived stress’ is a subjective construct of the stress experienced by employees and it appears to be associated with feelings of depression, sleep disorders, psychosomatic stress and complex psychosomatic stress. The way in which employees perceive stress factors may mediate the impact of job stress on the employee. In a study by Lee *et al* of nursing staff in Korea^30^ the perceived stress mediated the effect of job stress, resulting in the development of depression among the employees. A future study could clarify the mechanism of the effect of perceived stress on factors related to job content.

The limitations of the present study include a small sample size, which affects the generalisation of the results to other teams of employees in relative structures. The cross-sectional nature of the study highlighted correlations between variables which are not all necessarily causative. It is probable that there are other indirect variables that may have a stronger acting role (level of education, earnings, possibilities of progress, conflict of roles, quality of collaboration between personnel, etc.). Finally, lack of a control group limits the significance of the findings. Future studies should take into consideration the methodological limitations of the present study and extend the results related to job stress and burnout.

The results of the present study highlight the need for a stress management training programme in an in-patient facility such as the Leros Asylum. This is very important keeping also in mind the particular framework in the Leros Asylum (lack of trained personnel and educational material). Despite the enduring financial recession in Greece, we strongly believe that in work environments such as Leros PIKPA Asylum the application of programmes for stress management and the training of the nursing staff in new models of nursing care can significantly help the majority of the personnel.
